# Cancer of the Tongue

**Published:** 1890-01-11

**Authors:** 


### SURGERY.

### CANCER OF THE TONGUE.

In one of his latest memoirs, Professor Volkmann presented an analysis of all the operations for the removal of cancer of the tongue performed by himself since the introduction of antiseptic procedures into the hospital at Halle. The immediate results have been highly satisfactory, only two out of ninety-one patients having died from the effects of the operation, and in one of these the wound had entirely healed. He had never seen suppurative cellulitis or erysipelas, or, indeed, any pyæmic complication, and he had much simplified the operation, abandoning ligature of the lingual arteries, tampons over the glottis, etc. He had two methods of operating : (1) When the disease was limited to the tongue, he would grasp the tumour with the forceps, or in a wire noose, and, drawing it well forwards, or towards the jaws, remove it with knife or scissors. The wire noose permitted the use, if it seemed expedient, of the electric cautery. In such cases he never incised the cheek. (2) When, however, there was extensive infiltration of the surrounding structures, he adopted a modification of Langenbeck's operation, which he preferred to Bilroth's. Dividing the jaw with a saw at a point between the bicuspid and first molar teeth, in the line of incision from the angle of the mouth to the larynx ; afterwards uniting the bones by stout silver wire, union, either truly osseous or a pseudo-urthrosis, following. In either operation the patients were chloroformed sitting in a dentist's chair. The remote or ultimate results of these operations were, however, not encouraging. Of the 35 cases of the major operation six could not be traced, 16 died of recurrent disease and eight from other causes, the cancer having also re-appeared. Three only are known to be still living, of whom two have had a return, and only one, a man 70 years of age, operated on six years ago, has remained perfectly well. Of the 56 minor operations, 19 have been lost sight of, 21 recurrents have died of cancer, and nine of other causes, while seven live still free from any return for from three to seven years.

